# Time to Reconsider Stem Cell Induction Strategies

**DOI:** 10.3390/cells1041293

**Published:** 2012-12-17

**Authors:** Hans-Werner Denker

**Affiliations:** Lehrstuhl für Anatomie und Entwicklungsbiologie, Universität Duisburg-Essen, Universitätsklinikum, Hufelandstr. 55, D-45122 Essen, Germany; E-Mail: hans-werner.denker@uni-due.de; Tel.: ++49-201-403792

**Keywords:** stem cells, pluripotency, bypassing pluripotency, direct reprogramming, tetraploid complementation, developmental potential, ethics, patenting

## Abstract

Recent developments in stem cell research suggest that it may be time to reconsider the current focus of stem cell induction strategies. During the previous five years, approximately, the induction of pluripotency in somatic cells, *i.e.*, the generation of so-called ‘induced pluripotent stem cells’ (iPSCs), has become the focus of ongoing research in many stem cell laboratories, because this technology promises to overcome limitations (both technical and ethical) seen in the production and use of embryonic stem cells (ESCs). A rapidly increasing number of publications suggest, however, that it is now possible to choose instead other, alternative ways of generating stem and progenitor cells bypassing pluripotency. These new strategies may offer important advantages with respect to ethics, as well as to safety considerations. The present communication discusses why these strategies may provide possibilities for an escape from the dilemma presented by pluripotent stem cells (self-organization potential, cloning by tetraploid complementation, patenting problems and tumor formation risk).

## Abbreviations

EB: embryoid body
EpiSC: epiblast stem cell
ESC: embryonic stem cell
EU-CJ: European Court of Justice
hESC: human embryonic stem cell
hiPSC: human induced pluripotent stem cell
iPSC: induced pluripotent stem cell
IVF-ET: *in vitro* fertilization and embryo transfer
mESC: mouse embryonic stem cell
miPSC: mouse induced pluripotent stem cell
PS: primitive streak
TC: tetraploid complementation

## 1. Introduction

Recent developments in stem cell research suggest that it may be time to reconsider the current focus of stem cell induction strategies. During the previous five years, approximately, the induction of pluripotency in somatic cells, *i.e.*, the generation of so-called ‘induced pluripotent stem cells’ (iPSCs), has become the focus of ongoing research in many stem cell laboratories, because this technology [1] promises to overcome limitations (both technical and ethical) seen in the production and use of embryonic stem cells (ESCs). A rapidly increasing number of publications suggest, however, that it is now possible to choose instead other, alternative ways of generating stem and progenitor cells bypassing pluripotency. These new strategies may offer important advantages with respect to ethics, as well as to safety considerations. This will be discussed in the present communication.

A common characteristic of ESCs, as well as iPSCs, is *pluripotency*. Until recently, a widely held opinion was that this property is a precondition for the desired combination of proliferative capacity and differentiation potential of the cells. Therefore, although the final interest of researchers is typically not in pluripotency itself, but in obtaining sufficient quantities of progenitors and differentiated cells, most investigators have considered it essential to start their protocols with generating some sort of pluripotent cells (and to derive from them the cell type of interest secondarily). According to an impressive number of recent publications, however, this assumption does not seem to hold anymore: these studies report success in deriving multipotent stem and progenitor cells *without* passing first through a state of pluripotency. The alternative new strategies are being addressed using terms like “*direct reprogramming*” or “*bypassing pluripotency*”, and they appear to allow for directly generating *multipotent* stem or progenitor cells, which can then be differentiated to the desired mature cell state. Contrary to earlier belief, these alternatively derived cells appear to be easily expandable *in vitro*, at least in some cases [2,3,4,5,6,7,8,9,10,11,12,13,14,15]. 

One of the reasons why these alternative strategies are being developed is that they may reduce the risk of *tumor formation* after cell transplantation, a risk that is of concern in any use of pluripotent cells (reviewed in [16]). Here, I will concentrate on *ethical* problems that are likewise connected with the generation and use of pluripotent cells, including aspects of patentability that have become apparent in a recent ruling of the European Court of Justice (EU-CJ) [17]. I will argue that taking the potentiality of cells into focus appears very timely and obviously needs to be pursued seriously when considering not only safety aspects (tumor formation risk), but also stem cell ethics. The new, alternative strategies of stem cell derivation now seem to open a chance for circumventing the ethical/patenting problems, if these protocols indeed allow to safely bypass pluripotency. This would provide a strong argument for generally preferring this new type of strategy in future stem cell research policy. 

## 2. Ethical Implications of Pluripotency

In current literature, it has become customary to address human iPSCs (hiPSCs) as ethically non-problematical without contemplating this any further (example: “*The use of such cells…circumvents the ethical issues associated with human cells*” [18]). This shorthand classification reflects the fact that, in contrast to ESCs, iPSC derivation does indeed not involve sacrificing embryos. On the other hand, statements that certain ethical problems are still connected even with iPSC technology can also be found; they are so far relatively rare, however [19,20]. The reason for such warnings is rooted in peculiar properties that are common to ESCs and iPSCs and that are connected with their pluripotency. I will discuss these implications here in the context of two scenarios: (1) self-organization potential (embryoid body formation); and (2) direct cloning potential (tetraploid complementation). 

### 2.1. Self-organization Potential: Totipotency *vs.* Omnipotency and Pluripotency

A characteristic property of all pluripotent cells is their ability to form *“embryoid bodies” (EBs)*, a phenomenon that is best known from suspension cultures of mouse ESCs [21]*.* Of practical interest is that formation of these embryo-like structures promotes the formation of germ layers. An aspect that has been studied much less intensely is, however, what degree of order the germ layers and their derivatives can attain in EBs, *i.e.,* in particular the question how close their organization can come to the basic body plan of viable embryos. Recently, this aspect appears to receive increased attention. 

Already in one of the pioneering papers on ESCs, Thomson *et al.* [22] reported on the spontaneous formation of astonishingly embryo-like structures in dense cultures of common marmoset ESCs (Callithrix jacchus, a South American primate); the structures they observed were described to consist of a flat embryonic disc as typical for primates, with an apparently well-organized primitive ectoderm (epiblast), primitive endoderm and even an amnion with amniotic cavity, a yolk sac. Ethically, most relevant is that those authors depicted and described, within this embryonic disc, an area of ordered ingression of cells, which they remarkably addressed as a *primitive streak (PS).* The PS plays a key role in vertebrate development: on one hand, it is the site where the formation of the definitive germ layers takes place (it is the site of the ingression of mesoderm and definitive endoderm); on the other hand, the PS is also instrumental in individuation. The anterior part of it is the equivalent of Spemann’s organizer, which plays a central role in laying down the *basic body plan*, *i.e.,* the ordered arrangement of germ layers and their derivatives according to the main body *axes* (specifically the anterior-posterior = cranio-caudal axis and right-left asymmetry). The relevance for individuation becomes obvious when we remember that development of single or double organizers is decisive for the formation of a singlet *vs.* monozygotic twins (discussed in [23]). The decisive role that the organizer and the entire PS play in individuation has been the basis for legal regulations concerning the time frame for permissible research on human embryos (e.g., the limit set at the 14^th^ day of development, in some countries, like the UK).

The report by Thomson *et al.* [22] remained unique for many years: a comparably high degree of order (in the sense of a basic body plan) was not reported to develop in ESC cultures in any other species, including the rhesus monkey and the mouse. In spontaneously developed human teratomas, a morphology coming astonishingly close to that of early post-implantation stages has occasionally been found (see [23]). Recently, however, the situation appears to have changed: locally restricted gastrulation-like events have been detected to occur more regularly than previously thought in EBs formed *in vitro* [24,25,26,27,28,29]. These observations suggest that the degree of order attained during ESC ‘gastrulation’ *in vitro* is/can be much higher than most people assumed at earlier times, and consequently, self-organization and axis formation phenomena in embryoid bodies have now become a topic for ongoing research [25,27] with a focus on gastrulation and primitive streak formation. Remarkably, in a number of cases, authors now directly compare this with *in vivo* embryogenesis and detect in these *in vitro* phenomena parallels to the formation of an anterior–posterior axis (“anterior, middle and posterior primitive streak”) *in vitro* [24,27,29]. The degree of order that can develop in primitive streak equivalents *in vitro* was considered remarkable and astonishing by the authors of one of these recent papers [27]. Retrospectively, the observation by Thomson *et al.* [22] about the formation of impressively well-structured embryonic anlagen with a ‘primitive streak’ in dense cultures of marmoset monkey ESC now does not appear so exceptional or even unthinkable anymore (as it had been considered to be by some commentators, at least at the peak of the ESC ethics debate in Germany [30]). However, from the developmental biology point of view, there are reasons why such pattern formation potential can indeed be expected to be present in ESC colonies [23]. 

It must be emphasized that such a phenomenon of early embryonic morphogenesis *in vitro* (specifically: more or less ordered gastrulation) has never been observed in comparable cultures of non-pluripotent cell types (e.g., epithelial cells, fibroblasts) and, thus, obviously primarily depends on *intrinsic properties of pluripotent cells*. Importantly, the development of structural order in cultures of pluripotent cells does not appear to depend on specific instructions from the outside: it is seen in suspension cultures, *i.e.*, without the addition of other cell types and without attachment, as well as in dense flat cultures on feeder cells (fibroblasts), which likewise cannot be expected to provide specific patterning information. It is thus reasonable to assume that the morphogenetic processes observed are primarily *autonomous* and can be correctly addressed as processes of *self-organization* [25,27]. 

The biological basis for such self-organization phenomena has been discussed earlier [23]. Of specific interest is what role extra-embryonic cell types might play here. It is now well-established that in embryogenesis signal exchange between extra-embryonic (primitive/anterior visceral endoderm, trophoblast) and embryonic (primitive ectoderm/epiblast) cells is instrumental in laying down the basic body plan by providing asymmetry cues for the positioning of main body axes (for recent reviews, see [31,32]). Consequently, only stem cells that are able to differentiate into both embryonic and extra-embryonic lineages can be expected to possess the early embryonic pattern formation potential discussed here. In earlier years, some authors had argued that ESCs could not be considered *totipotent* because of defects in their capacity to differentiate the extra-embryonic cell types mentioned. However, this was incorrect: ESCs do have the capacity to differentiate extra-embryonic endoderm cell types in the mouse and to form these as well as trophoblast in primates (including the human) [33,34,35,36,37,38,39,40,41,42,43,44]. In fact, evidence for trophoblast formation in ESC cultures had indeed already been presented in the pioneering publications on non-human (rhesus and marmoset monkey) and human ESCs (reviewed in [41]). 

We should thus be aware that all cell types necessary for early embryonic development are present in colonies of pluripotent cells (or at least they can be present, depending on culturing conditions). Obviously, the term *“pluripotency”* does not describe the potential of these cells optimally, because they can form not only *many* (as the term suggests), but *all* cell types. Consequently, it has been proposed to use the term *“omnipotency”* to characterize this latter, comprehensive type of potential [45]. As far as *“totipotency”* is concerned, many authors use this term in the same sense as “omnipotency” (or “pluripotency”). In order to be more specific, it has been proposed to avoid the confusing term “pluripotency” and replace it by “omnipotency” for cells with the full spectrum of progeny diversification capacity and by “totipotency” for those which in addition can build a basic body plan, *i.e.*, have individuation capacity [45] (Table 1). However, such a strictly defined terminology is still not being used commonly. Also it is still unclear which detailed structural/molecular properties would be responsible for determining these differing potentialities of the cells. It has been assumed that the quality that totipotent cells (like blastomeres of early embryos) must possess in addition to their omnipotency might be some cytoplasmic asymmetry cues instrumental in laying down future body axes (basic body plan, discussed in [23]). However, whether detectable asymmetry cues do indeed exist in totipotent early embryonic cells (zygote, blastomeres) is still a matter of discussion. The observations reviewed above about the self-organization potential of “pluripotent” cells *in vitro* [24,25,26,27,28,29] (as well as the phenomenon of twinning [23]) teach us that, if such asymmetry cues exist, they are dispensable and can be replaced by extrinsic (including stochastically arising) asymmetry signals in cultures of “pluripotent” cells. This makes it appear questionable whether any clear line can indeed be drawn between “omnipotency” and “totipotency”.

**Table 1:** Terms Used to Describe Stem Cell Potentiality.

Term	Definition	Examples
**Unipotency**	Potential to generate one specific cell type	Spermatogenic stem cell
**Multipotency**	Potential to generate multiple but not all cell types	Mesenchymal stem cell and some other somatic/ adult stem cell types
**Pluripotency / Omnipotency**	“Pluripotency” is widely used to describe potential to generate all cell types, but is linguistically incorrect and misleading (*plures =* several; *omnes* = all). Should thus better be *replaced by* Omnipotency*.*	
***Subgroups:***		
“Restricted” Pluripotency / Omnipotency (“primed state”)	Potential to generate all cell types, but **no** potential for chimera formation, **no** direct cloning capability (TC), **no** potential for early embryonic self-organization (basic body plan) in EBs (?).	EpiSC (some) hESC*(some) hiPSC*
“Full” Pluripotency / Omnipotency (“naïve state”)	Potential to generate all cell types, **plus** potential for chimera formation, direct cloning by TC and early embryonic self-organization (basic body plan) in EBs.	mESC, miPSC(some) hESC/hiPSC*“reverted” hESC/hiPSC*Marmoset ESC
**Totipotency**	*“Full” Pluripotency/Omnipotency* plus positionalcues for axis formation (self-organization, basic body plan)	ZygoteBlastomeres

**Note: **Some authors use the term *totipotency* for what is defined here as *pluripotency/omnipotency. * Indeed, no sharp line can be drawn between totipotency and pluripotency: The (probably cytoplasmic) positional cues for axis formation present in zygotes and blastomeres can be replaced in pluripotent/omnipotent stem cells by external factors. * For human and non-human primate ESC and iPSC, data on chimera formation and TC capabilities are lacking due to ethical problems. Assumptions on the presence or absence of these capabilities are thus speculative and rely on extrapolation from gene expression profiling data and growth factor requirements, which are somewhat similar to those of EpiSC. Some hESC lines show a bias in their differentiation capabilities, which has been interpreted as indicative of a “primed state”.

Realistically, it cannot be expected that the widespread use of the term “pluripotency” for ESC and iPSC will change easily. In the following, I will use the term pluripotency without quotation marks, *i.e.,* as it is mostly used in current literature. On the other hand, there may be reason to subdivide pluripotency/omnipotency into two subgroups in order to account for the fact that certain relevant properties are known to differ between individual cell lines [46] (Table 1). For mouse ESC and iPSC, it is well documented that, e.g., the potential for chimera formation, as well as the capacity for tetraploid complementation (to be discussed below), differ, depending on the cell line; such data are missing for hESC, however. 

In recent literature, two additional terms are in use: “naive” and “primed” states of pluripotency [47,48]. While the “naive” state would be typically represented by mouse ESCs (mESCs), the “primed” state would be found in mouse epiblast stem cells (EpiSCs); human ESCs (hESCs) would in their molecular and biological characteristics be more close to the latter (Table 1). The fact that various hESC lines show a bias to differentiate in a certain direction has been interpreted as an indication for their “primed” state [46,48]. Autonomous differentiation and self-organization have not been studied in detail in EBs, with regard to this distinction. Participation of cells in chimera formation has been checked for mouse EpiSC. Since the latter were found not to integrate well into host embryos, probably due to their epithelial phenotype, and because hESC and mouse EpiSC show related gene expression profiles, some authors assume that hESC would also lack chimera formation potential if tested. However, data proving this are missing due to the ethical problems associated with performing chimera formation experiments in the human. Since ethically relevant conclusions have been derived from these extrapolations, I will come back to this point further below when discussing tetraploid complementation.

Notwithstanding the fact that terminology is still changing in this rapidly progressing field, and that it tends to be confusing for ethicists and politicians, the ethically most relevant aspect to be considered must be seen in the self-patterning/self-organizing capacities, which are indeed an impressive peculiarity of these versatile cells, at least of their “naive” variants. We should also keep in mind that this self-organization is not restricted to *early* embryonic patterning/individuation phenomena, but is well documented for the later events of the formation of organ anlagen in cultures of pluripotent cells. These phenomena are used as *in vitro* models for experimentally studying developmental processes [49,50,51,52]. In conclusion, the fact that an inconsistent terminology is used by various authors should not divert our attention from the main fact, *i.e.,* that we are dealing here with cells that possess a very peculiar property, the capacity for self-organization, which can be expressed whenever these cells are growing in clusters.

Do these observations on morphogenetic processes in EBs have any ethical relevance? Pluripotent cells obviously possess *gastrulation* potential and can show impressive early embryonic pattern formation (*self-organization*) potential *in vitro*. These processes are central elements of *basic body plan* formation and *individuation* during embryogenesis [23]. As shown recently and discussed above, differentiation of EBs *in vitro* can come much closer to *in vivo* embryogenesis than originally thought by many. On the other hand, under the *in vitro* culturing conditions, which are routinely used, EBs rarely reach the high degree of order of a harmonious basic body plan. It is well known from developmental biology that during basic body plan development the degree of order attained depends very much on physical conditions [23]. In all known experimental systems, this is strongly influenced by any other (non-stem) cells or matrix simultaneously present [28]. As a consequence, early embryonic self-organization may or may not occur in a given situation *in vitro*, depending on culturing conditions. It is practically difficult if not impossible to safely exclude the initiation of self-organization processes in cultures of pluripotent cells. 

In summary, depending on the degree of stringency one likes to use with respect to ethical norms, it could be argued that EB formation does or does not offer arguments with respect to ethical aspects of pluripotent stem cell use in the human. Taking into account the current focus of stem cell research, it is of much interest that all these considerations do not only apply to ESCs but also to iPSCs, because the latter show the same biological properties, including EB formation capacity (although morphogenetic processes have so far been studied much less in detail in EBs formed from iPSCs). Ethically very relevant is that this morphogenetic potential is in case of ESCs an (unwanted) relic from the original cell source (embryo), whereas in the case of iPSCs, this potential is a new acquisition resulting from the stem cell induction strategy used and must thus be considered man-made.

### 2.2. Direct Cloning Potential: Tetraploid Complementation

*Tetraploid complementation* *(TC)* is a technology that allows to produce viable embryos and even offspring derived entirely from pluripotent stem cells that had been propagated before *in vitro* [53,54,55]. It is in use in many laboratories as part of gene targeting protocols, as well as stem cell quality testing, in the mouse. Although TC currently does not appear to be in use with human cells, the principal availability of this technology has ethical implications that are just starting to be recognized and must have consequences for future legislative actions and patenting regulations. This will be discussed in the following paragraphs.

Originally, TC was developed as a variant of chimera formation. The peculiarity of this technology is that pluripotent cells are combined not with diploid, but with tetraploidized blastomeres or, alternatively, are injected into tetraploid blastocysts. The individuals so produced are composed entirely of derivatives of the pluripotent cells of origin, whereas the tetraploid helper cells contribute only to the formation of extraembryonic tissues (placenta, yolk sac). Remarkably, cloning of viable mice by TC is successful not only with ESCs, but also with iPSCs. In the latter case, the term *“all-iPSC mice”* has become popular for the products of this type of cloning [56,57,58,59,60]. 

Testing of mouse cells for TC capability is often addressed as the “*gold standard*” of pluripotency, and its application is indeed being advocated in a way that might suggest using it also with human cells [56,58]. Should we indeed suppose that TC would work in the human if ever attempted? Some argue that TC will not be possible in the human, because hESC and hiPSC may represent a “primed state” of pluripotency not allowing chimera formation and TC, in contrast to the mouse counterparts in which this is successful, since mESC and miPSC represent the “naive” state [47,48] (see above; Table 1). Currently, there is no literature on any attempts at testing TC efficiency in the human (it may be asked whether this simply reflects the fact that such experiments have been deemed unethical [19]). However, even if we assume that TC would not work with hESCs and hiPSCs, this would not argue against the need to contemplate ethical implications: it has been shown that “primed” cells can be induced to revert to the “naive” state (and *vice versa*) [61]. Therefore, the pluripotent cells must be expected to possess a potential to allow TC, at least after conversion. 

Why could anyone possibly be interested in applying TC not only in the mouse, but with *human* cells? At the first sight, it may appear improbable that TC will be used for *reproductive cloning* in the human in any foreseeable future, since, so far, there is widespread consensus to consider human cloning unethical. However, there is reason to question whether this consensus can be expected to hold for long and worldwide. Even in the Western world, it has already been proposed to consider using TC technology in order to increase success rates in *in vitro* fertilization/embryo transfer (IVF-ET) clinics [62]. What those authors proposed in detail was to derive hESCs from IVF embryos, expand them *in vitro* and (re-) construct from them a (larger) number of embryos by TC. The necessary helper cells could possibly be trophoblast cells derived from hESC cultures (although it is still unclear whether this approach would work), so that helper embryos would not be needed. The numerous genetically identical embryos so produced would then be available for embryo transfer (while aliquots of the ESCs, as well as some of the embryos could of course be stored in liquid nitrogen to be available for later use in repeated attempts) [62]. Since this means cloning, and since reproductive cloning in the human is illegal, at least in a number of countries, one might be skeptical whether this strategy will ever be applied in IVF-ET in Western world countries. However, it cannot be excluded that legislation may develop in a different direction in other cultural environments. As an example, Buddhist authorities have expressed that they would consider it unethical to sacrifice human embryos in the course of “therapeutic cloning” (for the production of ESCs), but not so the (re-)construction (and cloning) of embryos as part of assisted reproduction (for literature see [63]).

While any application of TC for reproductive cloning in the human may appear improbable at this moment, it is already widely in use in *research* and *quality testing* with mouse cells, and the extension of this practice to human cells might appear logical. TC is recommended as the most rigorous pluripotency test (“gold standard“) for iPSCs in the mouse (examples: “*We therefore consider the tetraploid complementation as the state-of-the-art technique to assess the pluripotency of a given cell line*” [64]; “*This study underscores the intrinsic qualitative differences between iPS cells generated by different methods and highlights the need to rigorously characterize iPS cells beyond in vitro studies.*” [65]). Likewise, in the first reports on the generation of viable mice from iPSCs, it had already been suggested indirectly to apply TC technology for iPSC quality testing also in the human, for the very reason that this is considered the most rigorous pluripotency test [56,58]. Remarkably, it appeared necessary to refute this (implicit) recommendation for ethical reasons in a comment published subsequently [19].

On principle, there is indeed good reason to ask for stringent quality testing in iPSC research. Individual iPSC lines are observed, in the mouse, as well as in the human, to vary with respect to differentiation capacities, gene expression patterns and epigenetic marks/memory [38,66,67,68,69]. Stadtfeldt *et al.* [60] provided a typical and illuminating example. They observed that transcripts encoded within the imprinted Dlk1-Dio3 gene cluster were aberrantly silenced in most of the iPSC clones and that these clones failed to support the development of entirely iPSC-derived animals (“all-iPSC mice”) when TC was performed, thus revealing a lack of “complete/full pluripotency”. This failure could, however, be corrected by a treatment with a histone deacetylase inhibitor, which reactivated the locus. Naturally investigators must wish to have a test available to monitor the success of this type of cell quality improvement. In addition to epigenetic peculiarities, even chromosomal aberrations and gene deletions have been observed in pluripotent stem cell lines in certain cases [70,71,72]. Such observations could obviously be seen as a strong argument for using the most stringent pluripotency test (*i.e.*, TC), also with human iPSCs, in order to select “optimal” cell lines and/or stem cell derivation protocols. 

Quality testing is of particular importance if application of the cells for *therapeutic purposes* (cell and tissue replacement) in the human is considered (see [73] as a recent example) and, likewise, whenever iPSCs are used for *disease modeling*. I will address the risk of tumor formation in cell replacement therapies further below, but let us take a look for a moment at disease modeling. In such model investigations, the basic gene targeting experiments are typically done in the mouse, often including TC technology (which of course poses no ethical problem as long as one remains in the animal model) (as an example, see [64]: “*Genetic manipulation of iPS cells in combination with tetraploid embryo aggregation provides a practical and rapid approach to evaluate the efficacy of gene correction of human diseases in mouse models.*”)*.* However, investigators may ask themselves how they can translate the observations made in the mouse model subsequently to human therapy without also testing human iPSCs with comparable stringency, applying a protocol that is comparable with that used before for the mouse cells within the same experimental project. Could this be considered a valid argument for the application of TC with human cells in order to use again the most stringent test? Would this argue for producing human TC embryos (“test embryos”) that might allow studying differentiation and gene expression *in vitro*? It should be seen that even without transferring such human “test” embryos created from iPSCs to a uterus, this procedure would (re-)create the problem of embryo destruction, which the original idea of iPSC technology intends to eliminate. It would, e.g., clearly be in conflict with the German embryo protection law (Embryonenschutzgesetz). 

It is open in which direction legislation will develop in this respect in the different parts of the world during the next years. If legislation will permit using such a quality testing strategy (including research cloning by TC), appropriate information that this would involve creation of human embryos would definitely need to become one part of the information to be given to cell donors when *informed consent* is obtained. At present, cell donors included in hiPSC production programs are not being informed about the (at least theoretical) possibility of cloning embryos by TC from the pluripotent stem cells to be produced, in no country of the world as far as I have been able to find out. Long-term banking of hiPSCs, which is presently being established in many countries, aggravates the problem: the principal availability of TC cloning technology will probably make it imperative for many cell donors to insist in establishing extremely stringent control over the use of the cells, even after long term storage (perhaps even beyond the personal death of the cell donor). Even though TC is not currently in use with hiPSCs, the principal availability of the technique already opens a completely new dimension for ethical requirements of cell banking due to the discussed potentiality aspects. I will not expand on aspects of informed consent here, because it has been addressed before [74], but it should be seen that appropriate regulations are still missing. In conclusion, the principal availability of TC technology confronts us with an ethical problem whenever human pluripotent cells are created or handled (ESCs or iPSCs).

### 2.3. Patenting and Pluripotency

The self-organization/individuation capacity, as well as the TC capability, of pluripotent cells discussed above are obstacles against patenting [75]. This problem is perhaps most obvious in the case of “fully pluripotent” human iPSCs: when, as a result of the induction process, cells acquire TC capability, this implies that the produced stem cells can now allow direct cloning of individuals. No matter whether talking about individuals *in statu nascendi* or whether these are allowed to grow to birth, the TC products would be genetically identical “twins” of the cell donor. Patenting of cells possessing this potential would mean patenting of major elements of personal identity. Cell donors must have an interest in maintaining a maximum of control over their personal genome, in particular over any propagation and distribution of it. Apart from the possibility of cloning, sexual reproduction is also a topic in this context: pluripotent cells (ESCs and iPSCs) are able to differentiate *in vitro* into germ line cells [76,77]. With regard to both scenarios, *i.e.*, asexual (cloning) and sexual reproduction, induction of pluripotency in somatic cells (which originally had been ethically neutral, like fibroblasts or epithelial cells) must thus be considered to create an ethical problem with respect to safe cell banking and/or cell distribution during scientific collaboration, to informed consent practice and to patenting. 

This problem and its implications for patenting regulations is just beginning to be recognized. So far, most interest has been focused on aspects of sacrificing embryos for the generation of ESCs. The *potentiality* of the cells, specifically the implications of inducing pluripotency, is a new theme that will have to be dealt with in future regulations. However, the recent ruling of the European Court of Justice (EU-CJ) mentioned in the Introduction [17] is already of interest in this regard: while its main focus was on the problem of sacrificing embryos, it appears remarkable that the Court’s ruling addressed potentiality in a somewhat novel way. The Court had been asked to position itself concerning definitions of the term “embryo” in the context of the legal/patenting regulations in question. It was ruled that “*any human ovum after fertilization, any non-fertilized human ovum into which the cell nucleus from a mature human cell has been transplanted, and any non-fertilized human ovum whose division and further development have been stimulated by parthenogenesis constitute a 'human embryo'.”* And* “(I)t is for the referring court to ascertain, in the light of scientific developments, whether a stem cell obtained from a human embryo at the blastocyst stage constitutes a 'human embryo' within the meaning of Article 6(2)(c) of Directive 98/44.*” [17]. 

Arguments presented before by certain authors in favor of patentability of hESCs had claimed that the degree of respect to be paid to the human embryos of origin (and the degree of legal protection given to them) should depend *inter alia* on the way how they had been created (natural fertilization *vs.* intracytoplasmic sperm injection or nuclear transfer technologies, artificial egg activation/parthenogenesis), on the actual location of the early embryo in question (within the female genital tract or *in vitro*) or whether the embryo was already implanted in the uterus or not yet. Some authors even proposed that not all such entities should be regarded as true embryos and for that reason should not necessarily receive the same degree of respect and protection. So, for example, Vrtovec and Vrtovec have argued with regard to ethical aspects of patenting of pluripotent stem cells that “*the exclusion from patentability is probably not justifiable for human totipotent cells that are produced outside the human body by (…) ‘techniques which human beings alone are capable of putting into practice and which nature is incapable of accomplishing by itself’*” ([78], p. 3028). This argument has immediately been rejected [79], but can still be heard. 

A logical consequence of the recent ruling of the EU-CJ, however, is that the potentiality that the cells/embryos possess and not the way how they have been created needs to be seen as a major point in ethical considerations and patenting. The Court directly refers to totipotency, a term that, however, is given a different meaning by different authors, as discussed above [45]. The definition used by the Court is a narrow one which, in agreement with developmental biology, takes as a criterion the potential to develop a basic body plan, but not the ability to continue organ development to functionality (live birth), since the Court included parthenotes into the list. Parthenotes suffer from epigenetic/imprinting problems that become obvious particularly at post-implantation stages, but they can be saved by specific genetic/epigenetic interference, at least in the mouse [80]. The Court thus classified parthenotes as embryos, notwithstanding their reduced viability and notwithstanding their (“unnatural”) mode of creation. What was considered decisive is the principal ability of parthenotes to develop a basic body plan, an organismic wholeness.

On the other hand, the Court leaved decisions whether an ESC (or an ESC colony) constitutes a 'human embryo' to the referring national court. This obviously must initiate a discussion whether the ability of pluripotent cells to initiate gastrulation in EBs, and the principal availability of techniques for “assisted development” of pluripotent cells (like TC) would have to be considered as ethically relevant (and thus relevant for patenting). Would pluripotent cells need to be regarded as non-patentable because they possess the unique potential to form viable embryos after TC (in contrast to, e.g., fibroblasts or mesenchymal stem cells that are lacking this potential)? My personal answer is: yes. In conclusion, future court decisions and legislative actions will certainly need to use potentiality as a focus, specifically with regard to iPSCs, since the main point in reprogramming is to endow originally ethically non-problematic cells with new potentiality. When pluripotency is being induced these new properties of the cells include, as discussed above, early embryonic pattern formation (self-organization, gastrulation, individuation) and TC capability, biological characteristics that they from then on share with early embryonic cells.

## 3. Alternative Stem Cell Derivation Strategies

As mentioned in the Introduction, recent literature suggests that alternative strategies are becoming available that allow stem and precursor cell derivation, while bypassing pluripotency [2,3,4,5,6,7,8,9,10,11,12,13,14,15]. Until only a few years ago, it was assumed by most authors that somatic/adult stem cells are not sufficiently expandable *in vitro* and that only pluripotent stem cells (ESCs and iPSCs) offer the advantage of growing well and of being able, in addition, to differentiate as desired for regenerative medicine. In the “*classical*” approach, iPSCs are created by transduction and overexpression or at least temporal activation of the “Yamanaka factor” genes Oct4, Sox2, Klf4 and c-Myc. For transplantation, disease modeling experiments or drug testing, the pluripotent cells so created are subsequently converted into multipotent progenitor cells and these to the various differentiated cell types of interest. This basic strategy has been more or less the same in all investigations involving the creation of pluripotent stem cells, irrespective of the cell type of origin chosen (fibroblasts, epithelial cells, *etc.*), and also irrespective of the mode of derivation (transient or permanent genetic or epigenetic modification). Since in this strategy the generation of pluripotent cells is the first goal (in the case of iPSCs, it is the induction; in the case of ESCs, the maintenance of pluripotency/omnipotency), it unfortunately creates exactly the ethical problem posed by the developmental potential, which we have discussed above. 

The *alternative strategies bypassing pluripotency* aim to directly induce tissue-specific stem and progenitor cells, while avoiding the activation of “pluripotency genes”. Direct reprogramming, not including a (transient) state of pluripotency, has now been described for the derivation of, e.g., cardiomyocytes, blood progenitors and neuronal cells, and in some of these cases, the derived cells were efficiently expandable *in vitro*, a property that many authors previously considered to be a specific characteristic and major advantage of pluripotent stem cells only. Literature on this new line of research is very rapidly expanding [2,3,4,5,6,7,8,9,10,11,12,13,14,15]. Indeed, these new strategies appear to open a chance to avoid the ethical (including patenting) problems posed by an early embryonic pattern formation/self-organization potential of cells (Table 2).

**Table 2:** Some Pros and Cons of Induction of Pluripotency *vs.* Direct Reprogramming.

		Pluripotent Stem Cells		Direct Reprogramming (to Uni- or Multipotency)
		ESC	iPSC	
**Ethical and Patenting Problems:**				
	Embryo destruction	+	0	0
	Cloning capability (TC)	+	+	0
**Tumor Formation Risk**		high	high	lower
**Time Required for Derivation**		long	long	shorter
**Growth/Expandability *in vitro***		high	high	varying (depending on cell type)
**Number of Cell Types Formed**		high	high	low

One of the incentives for why researchers are now trying to develop the new strategies of direct conversion of somatic cells to a stem/progenitor state, bypassing pluripotency, lie in the intention to reduce the risk of *tumor* formation after transplanting the derived cells. Tumor formation is of concern because on one hand, one of the basic properties of pluripotent cells is their ability to form teratomas (it is even a criterion for pluripotency) [16,81,82,83,84]. In addition, it is known that when ESCs are propagated *in vitro*, they tend to adopt properties of transformed cells in the same way as other types of cells do under such conditions, so that their difference from embryonic carcinoma (EC) cells becomes more and more indistinct [72,85]. Of specific concern is that the transcription factor genes activated in the course of iPSC induction either are known oncogenes (c-Myc) or have been shown to promote cell transformation [16,86]. Likewise, suppression of p53 is helpful in the generation of iPSCs, but increases genome instability (reviewed in [16,87]). There are indications that the tumor-formation risk is indeed reduced or eliminated with the new strategies [13,16], although we must keep in mind that this risk is generally not restricted to pluripotent cells [88,89]. 

A specific word of caution appears to be in place, however: in order for any such alternative strategy to be ethically acceptable, it must be made sure that it does not include passing through a transitory state of pluripotency that could remain undetected. There are indications for the occurrence of a rare transient cell population within ESC and iPSC cultures that express high levels of transcripts found in two-cell embryo blastomeres which are totipotent [90]. Many of the induction protocols require very long culturing time periods, and we are far from understanding exactly what cascade of events takes place during this time period. Apart from the activation of specific lineage genes, some of the protocols include activation of Oct4, whereas others omit it, some use combinations of certain (but not all) of the other Yamanaka factors, while others do not. Which of the possible protocols will be safest in order to exclude even a transitory gain of TC capability and of self-organization capacity? It will have to be discussed which genes should be seen here as crucial (e.g., genes involved in early embryonic pattern formation/self-organization processes) [63,91]. Research on early development in mammals has advanced to a stage at which it should be possible to select, as targets, genes involved in key developmental processes in individuation, e.g., signaling events involved in the formation of the body axes and in ordered gastrulation [32,92]. Gene expression profiling in stem cell cultures is to be appropriately combined with this [93]. These will be important topics for future research. 

## 4. Conclusions

As a consequence of the advent of alternative cell reprogramming technologies, bypassing pluripotency, we should consider it urgent to contemplate whether it is indeed still reasonable and defendable to produce stem cells with the maximal possible potential in the human. Should the direct induction of a reduced level of stem and progenitor cell properties be the preferable option from now on? As discussed above, the new strategies promise to offer solutions for both types of problems connected with pluripotency, the ethical problem (cloning potential), as well as the tumor formation risk. One may ask why such a redirection of focus (direct reprogramming) has not been searched for more actively already during previous years, although ethical arguments about why such efforts should appear mandatory had already been published [45,63,91,94,95].

At the present time point, the details of the optimal protocols for the induction of stem and progenitor cells with limited differentiation potential are still under investigation. In addition, it will be important to decide about strategies for testing the developmental potential of these alternatively derived stem and progenitor cells. Testing this will be critical in order to make sure these cells do not indeed possess the problematical properties of pluripotent cells (hESCs and hiPSCs), like clonability by TC and tumorigenicity. For ethical reasons, it cannot be defended to test the biological properties of hiPSCs by actual cloning via TC ([19]; this was already discussed for hESC testing by an Enquete Commission of the German Bundestag in 2003 [96]). Instead, appropriate combinations of *in vitro* gene expression profiling combined with *in vitro* culturing of the cells under conditions that avoid the initiation of individuation processes may be applied as a surrogate test in the sense of a first approximation [97]*.*

If the work which is ongoing in many laboratories on direct reprogramming to a stem and progenitor state with restricted potential continues to show success, we should ask ourselves whether it is indeed time to reconsider general stem cell strategies and policy. We should ask whether the widely preferred practice of activating the pluripotency program (*i.e.,* creating iPSCs) should now be replaced by relying on the new alternative strategies bypassing pluripotency in the human. The ethical considerations discussed in the present communication should be of particular interest for developing appropriate policies for stem cell banking for which many initiatives have started in a number of countries.
